# Caffeic and Dihydrocaffeic Acids Promote Longevity and Increase Stress Resistance in *Caenorhabditis elegans* by Modulating Expression of Stress-Related Genes

**DOI:** 10.3390/molecules26061517

**Published:** 2021-03-10

**Authors:** Sofia M. Gutierrez-Zetina, Susana González-Manzano, Begoña Ayuda-Durán, Celestino Santos-Buelga, Ana M. González-Paramás

**Affiliations:** 1 Grupo de Investigación en Polifenoles (GIP-USAL), Unidad de Nutrición y Bromatología, Facultad de Farmacia, Campus Miguel de Unamuno, Universidad de Salamanca, 37007 Salamanca, Spain; sofia_martinez@usal.es (S.M.G.-Z.); bego_ayuda@usal.es (B.A.-D.); csb@usal.es (C.S.-B.); paramas@usal.es (A.M.G.-P.); 2 Unidad de Excelencia. Producción, Agrícola y Medioambiente (AGRIENVIRONMENT), Parque Científico, Universidad de Salamanca, 37185 Salamanca, Spain

**Keywords:** caffeic acid, dihydrocaffeic acid, lifespan, oxidative stress, thermoresistance, *Caenorhabditis elegans*, IIS pathway

## Abstract

Caffeic and dihydrocaffeic acid are relevant microbial catabolites, being described as products from the degradation of different phenolic compounds i.e., hydroxycinnamoyl derivatives, anthocyanins or flavonols. Furthermore, caffeic acid is found both in free and esterified forms in many fruits and in high concentrations in coffee. These phenolic acids may be responsible for a part of the bioactivity associated with the intake of phenolic compounds. With the aim of progressing in the knowledge of the health effects and mechanisms of action of dietary phenolics, the model nematode *Caenorhabditis elegans* has been used to evaluate the influence of caffeic and dihydrocaffeic acids on lifespan and the oxidative stress resistance. The involvement of different genes and transcription factors related to longevity and stress resistance in the response to these phenolic acids has also been explored. Caffeic acid (CA, 200 μM) and dihydrocaffeic acid (DHCA, 300 μM) induced an increase in the survival rate of *C. elegans* under thermal stress. Both compounds also increased the mean and maximum lifespan of the nematode, compared to untreated worms. In general, treatment with these acids led to a reduction in intracellular ROS concentrations, although not always significant. Results of gene expression studies conducted by RT-qPCR showed that the favorable effects of CA and DHCA on oxidative stress and longevity involve the activation of several genes related to insulin/IGF-1 pathway, such as *daf-16*, *daf-18, hsf-1* and *sod-3*, as well as a sirtuin gene (*sir-2.1*).

## 1. Introduction

Phenolic compounds are important components of the human diet because of their ubiquity in plant-based foods. There is growing evidence that moderate intake of these compounds in the long-term may have benefits for human health in preventing or reducing the risk of different chronic diseases [1,2]. However, the actual contribution of these compounds to maintaining health and the mechanisms underlying their biological effects remain unclear. The activity of phenolic compounds has traditionally been linked to their antioxidant properties as related to their reducing power and free radical scavenging capacity. However, as progress has been made in understanding their bioavailability and metabolism, questions have arisen about the mechanisms of action really involved in the in vivo biological activity of these compounds [3].

In general, the absorption of phenolic compounds in the small intestine is low, so that they mostly reach the colon unchanged, where they interact with the gut microbiota being catabolized to a range of low molecular weight metabolites, which might be absorbed and distributed by systemic circulation. After uptake, the microbial metabolites can be additionally biotransformed, either in the enterocytes or liver, into conjugated forms (glucuronides, sulfates, and methylated derivatives) and distributed by systemic circulation [4,5]. The predominant catabolites generated by the microbiota from phenolic compounds are phenolic acids with up to three aromatic hydroxyls and a lateral chain from one to five carbons [5,6]. These phenolic metabolites might represent the main circulating forms of dietary phenolic compounds in the body and may be responsible for a part of the bioactivity associated with the consumption of the original compounds [7]. Nonetheless, the low concentrations of the metabolites that can be reached at both the plasma and tissues (nanomolar to low micromolar order), as well as the fact that the conjugated forms have less antioxidant capacity than the original compounds, call into question that the direct antioxidant effect can play a prominent role in the in vivo activity of phenolic compounds [8]. The most recent studies suggest that, at least in the case of flavonoids, the antioxidant effects in vivo could be more related to indirect mechanisms, due to the possibility of intervening in cell signalling processes, regulating gene expression at different levels [9].

**Figure 1:**
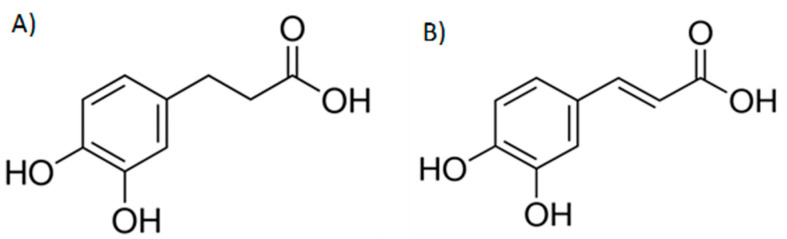
Chemical structures of dihydrocaffeic acid, DHCA (**A**) and caffeic acid, CA (**B**).

Caffeic and dihydrocaffeic acid (i.e., 3,4-dihydroxyphenylpropionic) (Figure 1) are relevant microbial catabolites, being described as products from the degradation of hdroxycinnamoyl derivatives (e.g., chlorogenic acids), anthocyanins, or flavonols [10,11]. Furthermore, caffeic acid is found both in free and esterified forms in many types of fruits (berries, kiwis, plums, pears or apples, among others), usually representing more than 75% of total hydroxycinnamic acid content, as well as in high concentrations in coffee [12]. 

In recent years, the nematode *Caenorhabditis elegans* has been used as a model organism to study the effects and subjacent molecular mechanisms of action of phenolic compounds, in processes such as oxidative stress, caloric metabolism, or longevity [13]. In previous studies of our group, different flavonoids, i.e., catechins, quercetin, and their phase II metabolites [14,15,16] and polyphenol-rich products [17,18,19], have been assessed for their effects on longevity and oxidative stress in *C. elegans*, demonstrating in general favorable outcomes in both respects. Furthermore, the molecular mechanisms behind the life-expanding and antioxidant effects of flavonoids have been evaluated by studying their influence on different genes and transcription factors of the insulin/IGF-1 signaling (IIS) pathway [20,21]. Thus, it was found that both epicatechin and quercetin showed modulating effects on this pathway, but not acting exactly in the same way and targeting the same genes.

With the aim of continuing delving into the effects and mechanisms of action of phenolic compounds, in this work, the influence of caffeic and dihydrocaffeic acids on lifespan and ability to counteracting oxidative damage are evaluated in *C. elegans*. Furthermore, the involvement of different genes and transcription factors related to stress resistance and longevity in the response to these phenolic acids has also been explored.

## 2. Results and Discussion

### 2.1. Stress Resistance Assays

**Figure 2:**
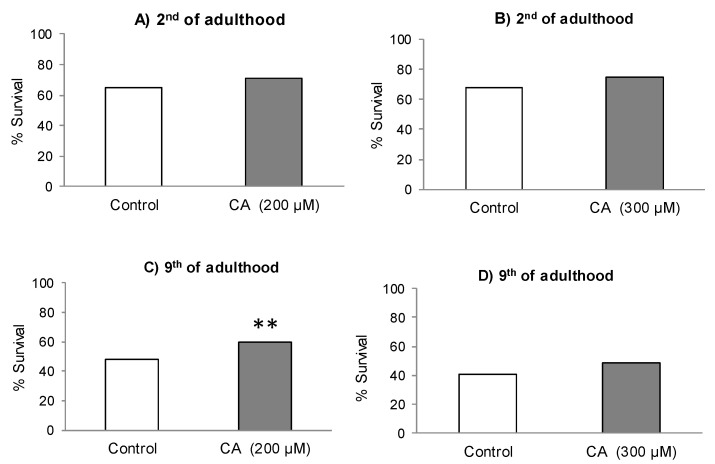
Survival percentages of *C. elegans* subjected to thermal stress (35 °C, 8 h) on the second and ninth day of adult, grown in the absence (controls) or presence of 200 µM of CA (**A**,**C**) or 300 µM of CA (**B**,**D**). The number of worms used in each assay was 300. The statistical significance was calculated using the Chi Square test (** *p* < 0.01 and *** *p* < 0.001).

Figure 2A–D shows the survival rates of control and CA-treated nematodes after subjected to thermal stress (35 °C, 8 h). In all cases, the cultivation in the presence of CA induced an increase in worm survival in relation to controls, although the difference was only significant in older worms (9th day of adulthood) treated with 200 μM (Figure 2C). Even though the differences were not significant (*p* < 0.05), greater relative increase in the survival was also found at day 9 in worms treated with 300 μM of CA. Pietsch et al. [22] also found a significant improvement in thermotolerance in worms treated with 200 μM of CA when the nematodes were subjected to thermal stress (35 °C, 8 h), with an increase in the survival rate of approximately 12% compared to control. Those authors also observed a significant enhancement in life duration of *C. elegans* with increasing CA concentrations from 100 to 300 μM, but a decrease at higher contents in the culture medium. Based on their and our observations, it appears, therefore, that CA concentrations of 200–300 μM in the culture medium could represent the inflection point from which the beneficial effects induced by this phenolic acid in *C. elegans* would begin to decline. Actually, Pietsch et al. [22] suggested the existence of a hormetic response to CA, so that its increase above certain levels would eventually produce a deleterious effect. A similar conclusion was obtained by our group in studies with quercetin-3,*O*-glucoside [23], which prolonged worm lifespan in a dose-dependent way at concentrations from 10 to 100 μM while decreased it at greater concentrations.

**Figure 3:**
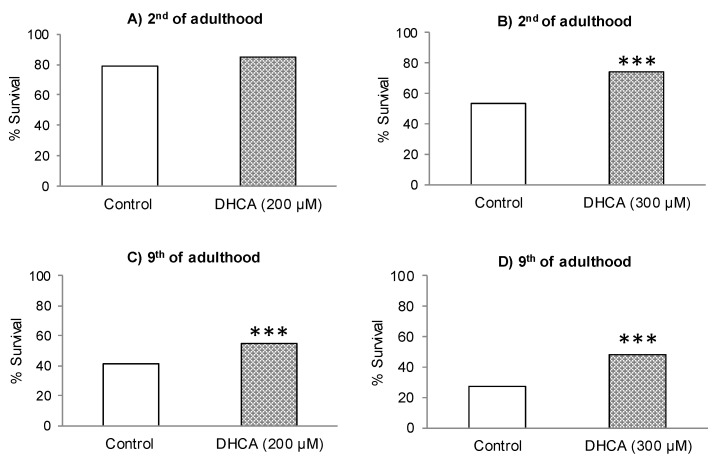
Survival percentages of *C. elegans* subjected to thermal stress (35 °C, 8 h) on the second and ninth day of adult, grown in the absence (controls) or presence of 200 µM of DHCA (**A**,**C**) or 300 µM of DHCA (**B**,**D**). The number of worms used in each assay was 300. The statistical significance was calculated using the Chi Square test (** *p* < 0.01 and *** *p* < 0.001).

Figure 3A–D shows the survival rates of nematodes not treated (controls) and treated with DHCA, after being subjected to thermal stress (35 °C, 8 h). Similar to CA, the treatment with DHCA at 200 μM or 300 μM led to an increase in worm survival. Nevertheless, in this case, the effect was not significant (*p* < 0,05) only in young worms (day 2) at 200 μM, while it is highly significant (*p* < 0.001) at 300 μM and in adults in post-reproductive stage (day 9), with respect to controls reproductive stage (day 9), with respect to controls.

The observations made might be interpreted as the stress protection capacity was improved by prolonged exposure to the assayed phenolic acids and/or older worms are more predisposed to their protecting effect. To the best of our knowledge, there are no previous studies on the effects of DHCA on oxidative stress resistance in *C. elegans*. Other authors have evaluated the effect of different caffeic acid derivatives on the response of the nematode to thermal stress. Thus, Havermann et al. [24] found that cultivation in the presence of 100 μM of caffeic acid phenethyl ester (CAPE) significantly increased the thermotolerance of worms submitted to a heat shock (37 °C, 8 h) at day 5 of adulthood. Similarly, Zheng et al. [25] observed a significant increase in the survival rate of *C. elegans* grown in a culture medium containing 50 μM of chlorogenic acid (i.e., caffeoylquinic acid) following thermal (35 °C) or paraquat-induced oxidative stress applied on the fifth day of adulthood.

The results obtained herein seem to indicate that DHCA is more efficient to improve the resistance against oxidative stress than CA, despite the presence of the double bond in the aliphatic side chain possibly being expected to provide greater H-donating ability and subsequent radical stabilization [26]. Actually, using different in vitro assays, other authors found no difference between the radical scavenging capacity of CA and DHCA [27] or even greater antiradical activity for DHCA than CA [28], suggesting that the antioxidant efficiency of CA and DHCA could depend on the system where the oxidation happens. Another implication of these observations is that not only could the intrinsic antioxidant ability be involved in the in vivo effects, but indirect mechanisms might also participate. Actually, nowadays, it is widely accepted that phenolic compounds exert their biological effects not only due to their role as conventional antioxidants, but also through their influence on cellular systems changing the expression of different genes by modulation of distinct transcription factors, acting simultaneously on various signaling pathways [29]. Among others, the improvement in the resistance to thermal stress would involve heat shock proteins (HSPs) [30], a type of molecular chaperone that helps to maintain proteostasis by destabilizing protein aggregates and promoting adequate protein folding [31]. In *C. elegans*, the regulation of the expression of these proteins may be mediated by the insulin signaling pathway [32,33].

### 2.2. ROS Levels

**Figure 4:**
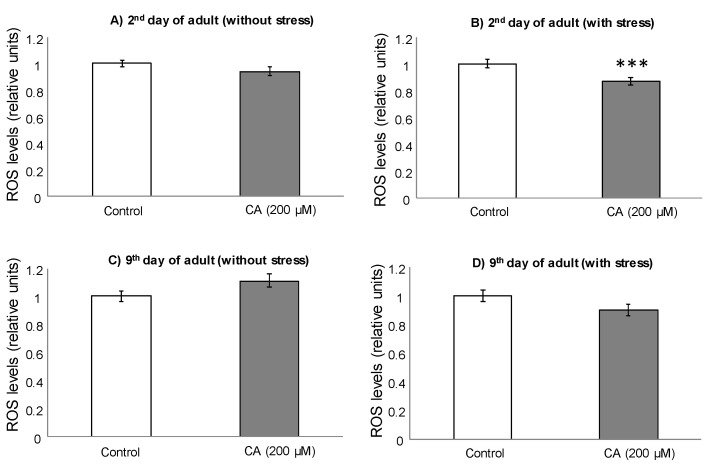
Relative intracellular levels of ROS, determined with DCFH-DA, in *C. elegans* on the second and ninth day of adulthood, cultivated in the presence or absence of CA (200 μM), not submitted (**A**,**C**) and submitted to thermal stress (**B**,**D**). The results are standardized with respect to control and represent the mean ± SEM. The statistical significance was calculated using ANOVA (*** *p* < 0.001).

To further delve into the effects of CA and DHCA in *C. elegans*, the intracellular levels of ROS were determined in control and treated nematodes. For these assays, worms were individually subjected or not to thermal stress (35 °C, 2 h) the second and ninth day of adulthood, after being cultivated in the presence of 200 μM CA or 300 μM DHCA. These concentrations were selected as treatment doses as they were those at which the more favorable effects on thermal stress resistance were found. At day 2, lower ROS levels were found in the worms treated with 200 μM CA compared to controls, although the decrease was only significant (*p* < 0.001) in the animals submitted to thermal stress (Figure 4A,B).

Somewhat different behavior was observed in the assay at day 9, where not significant differences (*p* < 0.05) were found between CA-treated worms and controls (Figure 4C,D). Nevertheless, a tendency to an increase in ROS levels was pointed in the nematodes treated with CA in the absence of thermal stress, while a decrease was noticed in those submitted to thermal stress.

**Figure 5:**
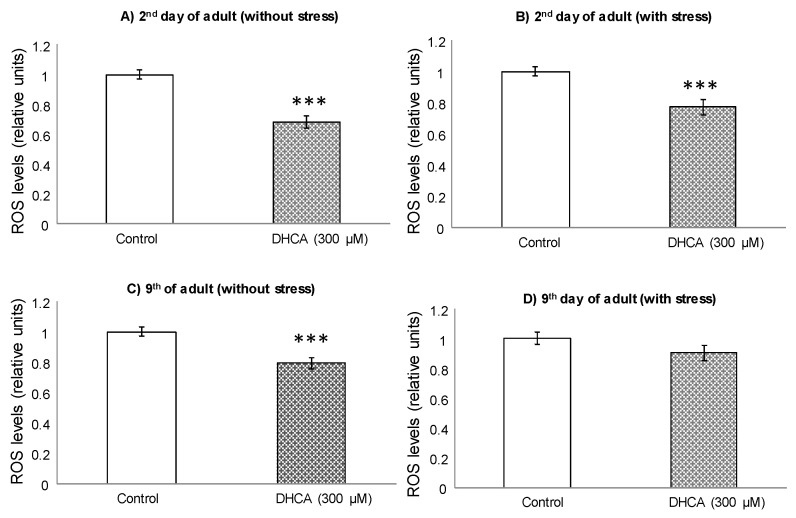
Relative intracellular levels of ROS, determined with DCFH-DA, in *C. elegans* on days 2 and 9 of adult, cultivated in the presence or absence of DHCA (300 μM), not submitted (**A**,**C**) and submitted to thermal stress (**B**,**D**). The results are standardized with respect to control and represent the mean ± SEM. The statistical significance was calculated using ANOVA (*** *p* < 0.001).

The treatment with DHCA 300 μM led to a decrease in ROS levels in relation to controls (Figure 5), which was significant (*p* < 0.001) in the absence of stress and in the measurements made at day 2, both under normal growing conditions and following application of thermal stress. As for CA, DHCA showed a more pronounced effect on decreasing ROS levels in younger animals, which might suggest that the ability to control ROS decrease with age.

In assays carried out in different cell lines, several authors also found that DHCA was able to act as a direct ROS scavenger and to increase eNOS activity in a dose-dependent manner [34,35,36]. Poquet et al. [37] demonstrated that DHCA decreased cytotoxicity and the production of pro-inflammatory cytokines in Ha-CaT cells, a model of keratinocytes, subjected to UV radiation, and related those effects to both a direct ROS scavenging activity and the improvement in endogenous antioxidant defenses. As far as we know, there are no previous studies about the influence of CA or DHCA on ROS levels in *C. elegans*. Nevertheless, Havermann et al. [24] evaluated the effect of CAPE and found that this caffeic acid derivative was able to significantly decrease ROS levels in young nematodes (day 2 of adulthood) when exposed to thermal stress (35 °C), while, under baseline conditions, it did not cause any change, which agrees with our observations for CA.

Regarding the results obtained herein, no clear relationship seems to exist between the observed effects of CA and DHCA on ROS levels and the ability to modulate resistance to oxidative stress. Similar conclusions were obtained in previous studies of our group for other phenolic compounds (e.g., epicatechin or quercetin), where the increased resistance to thermal stress was rather explained by an activation of endogenous defense mechnisms than by the anti-radical capacity of the compounds [20,21]. In this respect, modulation of the activity of antioxidant enzymes, along with the possibility of intervening in cellular signaling processes regulating expression of stress-related genes could be more plausible mechanisms to explain the effects of phenolic compounds than their direct antioxidant activity [9,29].

### 2.3. Lifespan Assays

**Figure 6:**
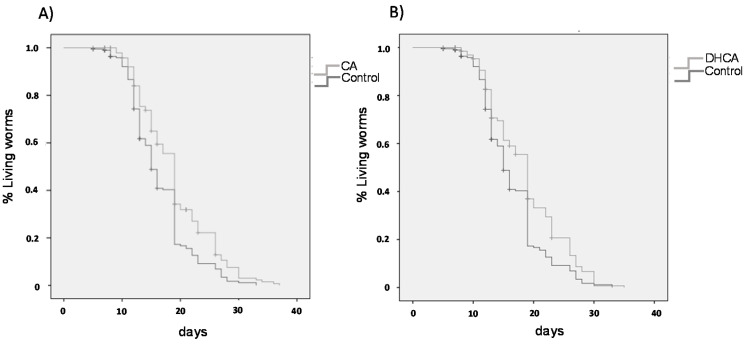
Survival curves of *C. elegans* cultivated at 20 °C in the absence (control) and presence of (**A**) CA (200 μM) or (**B**) DHCA (300 μM).

As for ROS measurement, the influence of CA and DHCA was evaluated using the concentrations in the culture medium that offered the most positive results in the stress resistance assays (200 μM and 300 μM, respectively). Figure 6 shows the survival curves of CA- and DHCA-treated and control worms grown under the same conditions, and Table 1 collects the calculated mean and maximum lifespan data, this latter determined as 10% of the longest-lived population. Similar results were obtained with both compounds, leaving to a significant increase (*p* < 0.001) in the mean and maximum life duration, compared to the control group.

The effect of CA on longevity in *C. elegans* was already studied by Pietsch et al. [22]. Similar to our results, those authors reported a significant increase in the mean life of CA-treated *C. elegans*. In their case, the assays were conducted with CA concentrations from 100 to 600 μM, finding a dose-dependent response, with the greatest effect for a concentration of 300 M, where a mean life increase of 11% was achieved compared to control, a similar value to that found in this study (+15%) for CA (200 μM). Havermann et al. [24] also observed significant increases in mean (9%) and maximum lifespan (17%) in worms treated with CAPE (100 μM) regarding control, whereas Zheng et al. [25] found that treatment with 50 μM of chlorogenic acid extended mean lifespan by 20.1%. As noted above, no studies have been found on the effects of DHCA on *C. elegans*. 

The increase in life duration found in the present study is in line with the improvement in the resistance to oxidative stress induced in the nematodes treated with CA or DHCA, and also coincides with the observations made by other authors working with different caffeic acid derivatives [22,24,25]. Actually, the existence of a relationship between increased stress resistance and longevity has already been noticed [38].

### 2.4. Analysis of Gene Expression by q-RT-PCR

**Figure 7:**
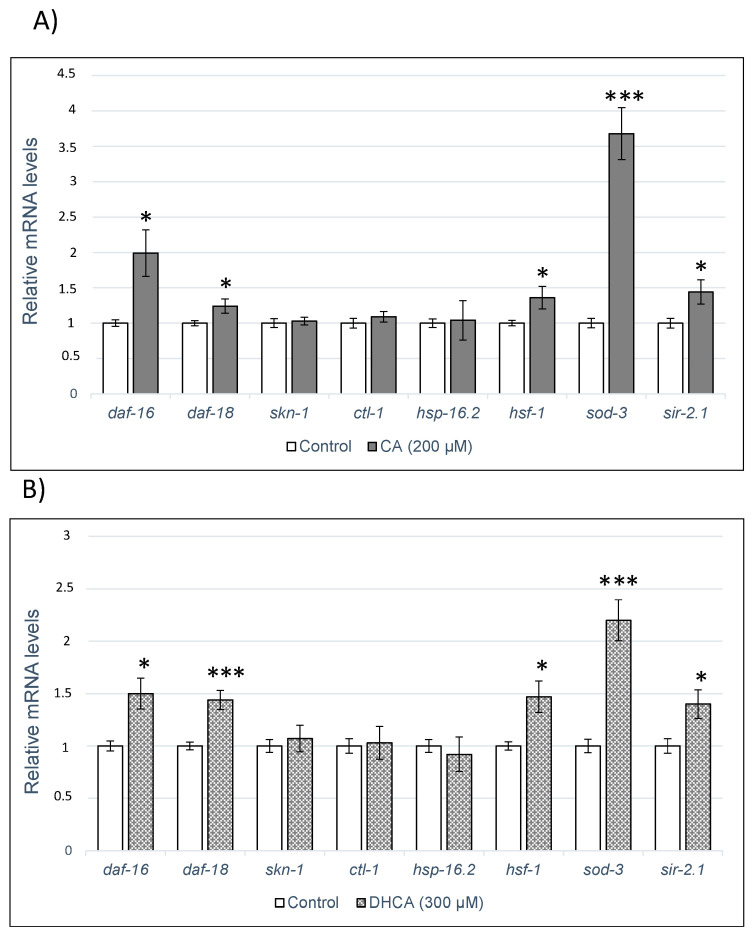
Expression of the genes daf-16, daf-18, skn-1, ctl-1, hsp-16.2, hsf-1, sod-3, and sir-2.1 in *C. elegans* cultivated in the absence (controls) and presence of (**A**) CA (200 M) or (**B**) DHCA (300 μM). Analyses were carried out by RT-qPCR, using act-1 as internal control. Nine separate experiments were always conducted and the results represent the mean ± SEM. The statistical significance was calculated using ANOVA (* *p* < 0.05, ** *p* < 0.01, *** *p* < 0.001).

In order to delve into the molecular mechanisms underlying the effects of CA and DHCA in *C. elegans*, their influence on the expression of several genes related to aging or response to stress (daf-16, daf-18, skn-1, ctl-1, hsp-16.2, hsf-1 and sod-3) and a sirtuin gene (sir-2.1) was evaluated using RT-qPCR. The results obtained are shown in Figure 7A,B. As it can be seen, no significant changes were observed in the expression of genes skn-1, ctl-1, and hsp-16.2 by the treatment with either CA or DHCA, while both compounds increased the expression daf-16, daf-18, hsf-1, sod-3, and sir-2.1, with respect to the control worms. The overexpression of those genes suggests that they could be involved in the enhancement in lifespan and stress resistance produced in the worm by CA and DHCA.

The daf-16 gene encodes the transcription factor DAF-16, the unique ortholog of the FOXO proteins family existing in *C. elegans*. Those proteins belong to the Forkhead group of transcription factors regulated by the insulin/PI3K/Akt signaling pathway, which play crucial roles in regulating genes involved in cellular proliferation, stress tolerance, and longevity. The involvement of DAF-16 in the effects of CA or related compounds in *C. elegans* was also concluded by other authors. Pietsch et al. [23], in studies with mutants lacking daf-16 function, found that this gene was necessary to explain the enhancing effect of CA on lifespan and thermal stress resistance of the worms. Similarly, Havermann et al. [24] observed that the treatment with CAPE increased the translocation of DAF-16 to the nucleus, which was indispensable for the lifespan extension induced by the compound in *C. elegans*. Zheng et al. [25] also found that this transcription factor was required for the beneficial effects on aging produced by chlorogenic acid. Similar observations were made with other phenolic compounds, for which DAF-16 was concluded to be involved in the favorable effects that they exert in *C. elegans* [20,21,39,40,41,42]. No previous papers have been found reporting the effects of CA or related compounds on daf-18, a gene that encodes a homologous lipid phosphatase to the PTEN tumor human suppressor. DAF-18/PTEN counteracts the activity of AGE-1/PI3K in the IIS route, negatively regulating the pathway and thus playing an important role in metabolism, development, and longevity [43].

The sod-3 gene is a direct target of DAF-16/FOXO and, therefore, the observed overexpression of SOD-3 following treatment with CA and DHCA could be related to the increase in the expression of DAF-16. SOD-3 is a member of the superoxide dismutase family of proteins that is orthologous of human SOD2 (mitochondrial superoxide dismutase 2) and catalyzes the dismutation of superoxide radicals into hydrogen peroxide and diatomic oxygen, conferring cell protection [44]. Thus, the increase in the expression of sod-3 could contribute to the control of the levels of reactive oxygen species, which are decreased in worms treated with phenolic acids (Figure 4; Figure 5). Other authors also reported an increase in the expression of sod-3 in *C. elegans* following treatment with flavonoids or phenolic-rich plant extracts leading to life extension and/or improved stress resistance [39,45,46,47].

Another gene that was significantly overexpressed (*p* < 0.05) by the treatment with CA and DHCA was hsf-1. This gene encodes the thermal shock transcription factor HSF-1 in *C. elegans*, which regulates the expression of various molecular chaperones and proteases in response to heat and other forms of stress [32]. Actually, HSF-1 overexpression has been reported to promote longevity in *C. elegans* in physiological conditions (without stress) [48,49], being related with various mechanisms regulating longevity, such as the IIS pathway, mTOR (mechanistic target of rapamycin), or caloric restriction, although the precise way it works is not fully elucidated. In studies on *C. elegans*, mutant strains lacking hsf-1 function, Zheng et al. [25] suggested the partial involvement of HSF-1 in the increase in lifespan induced by chlorogenic acid, owing to a non-significant increase in life extension continued to be observed in the mutant nematodes.

As mentioned above, a line of defense response against high temperatures and other forms of stress is the induction of thermal shock proteins. These include HSP-100, HSP-90, HSP-70, HSP-60, HSP-40, HSP-30, and small HSP (sHSP) [50]. Some HSP, predominantly ATP-dependent chaperones of the HSP-70 and HSP-90 families, show a constitutive expression and besides being involved in stress response have roles in the development [51]. Other HSP, mainly sHSP of the HSP-16 family, are induced and would work during thermal or oxidative stress [52]. Molecular chaperones like HSP-16.2 are transcriptional targets of DAF-16 and HSF-1 [32]; however, in the present study, no change in the expression of hsp-16.2 was observed despite both daf-16 and hsf-1 being overexpressed in the worms treated with CA and DHCA (Figure 7A,B). The behavior of different HSP (hsp-3, hsp-12.6, hsp-16.1, hsp-16.41, hsp-17 and hsp-70) in response to CA was evaluated by Pietsch et al. [22], observing a decrease in the expression of five of them, with only hsp-12.6 showing a not significant increase. Taking into account those findings and the results obtained herein, no clear role can be concluded for these molecular chaperones in the effects of CA or DHCA in *C. elegans*. Moreover, while it has been reported that the lifespan extension associated with overexpression of HSF-1 could be partly due to the regulation of the expression of HSP’s [32], Baird et al. [53] found that overexpression of HSF-1 in a transgenic strain of *C. elegans* extended the mean life without affecting its ability to trigger HSP expression. This suggests that the increase in lifespan associated with increased HSF-1 activity would not only be related to the positive regulation of heat shock response genes, such as hsp-16.2, but it presumably could also involve transcriptional regulation of other unidentified HSF-1 targets. On the other hand, the transactivation potential of HSF-1 may be limited by several regulatory mechanisms, such as inhibitory proteins like AKT, factor binding protein of HSF-1 (HSB-1), DDL-1, DDL-2, or integrin-linked kinase (PAT-4/ILK), which would dictate the context-dependent activation status of HSF-1 protein [54,55].

In *C. elegans*, sir-2.1 encodes the sirtuin homologous of SIRT1 in mammals, which responds to metabolic changes in the cellular environment, including nutrient availability, energy, and cellular stress [56]. The performed analysis showed that treatment with CA or DHCA significantly increased (*p* < 0.05) the expression of sir-2.1 (Figure 7A,B). It had already been shown that the overexpression of sir-2.1 in *C. elegans* increased mean life and that this extension required DAF-16/FoxO [57], and similarly Berdichevsky et al. [58] described that SIR-2.1 could act in parallel with IIS on a stress-dependent way by activating DAF-16 and thus extending lifespan. Martorell et al. [40] also reported that both DAF-16/DAF-2 and SIR-2.1 were involved in the extension of life and oxidative stress protection induced in *C. elegans* treated with a procyanidin-rich cocoa extract.

Pietsch et al. [22] also found that the SIR-2.1 factor was necessary to explain the increased lifespan and improved thermotolerance induced by CA in *C. elegans*. However, they made the same observation in mutants lacking daf-2 and age-1, suggesting that these genes of the IIS route were not required for the CA effect, which led them to conclude that CA would promote longevity through an SIR-2.1-dependent stress response pathway independent of IIS [22]. Xiong et al. [59] also reported that the protective effects induced in *C. elegans* by a black tea extract against thermal stress and UV radiation were associated with an increase in the expression of sir-2.1 and sod-3, but not of daf-16. However, the same group found that the treatment of *C. elegans* with the main tea catechin, epigallocatechin-3-O-galate, resulted in an increase in longevity depending on AAK-2, SIR-2.1 and DAF-16 [46]. All in all, while SIR-2.1 seems to be involved in the mechanisms leading to lifespan extension and protection against oxidative stress induced by different phenolic compounds in *C. elegans*, its interrelation with the IIS route is not clear and might differ depending on the compound considered.

Finally, no changes were found in the expression of the skn-1 and ctl-1 genes in the worms treated with CA or DHCA compared to controls, suggesting that they are not necessary to explain their effects on *C. elegans*. Havermann et al. [24] did not find either that SKN-1 was related to the longevity promoted by CAPE in *C. elegans*, nor did they observe translocation to the nucleus of this factor after treatment with the compound. It was also reported that *C. elegans* mutants who lacked skn-1 continued to present an increase in lifespan following treatment with quercetin [60,61] or phenolic-rich extracts from blueberries [62], indicating that SKN-1 was not necessary to explain longevity induced by those compounds. The skn-1 gene encodes the homologous of the mammal transcription factor Nrf-2 that regulates an array of detoxifying and antioxidant defense gene expression. SKN-1 is activated through translocation to the nucleus in response to oxidative stress, inducing the expression of its target genes by binding to the antioxidant response element (ARE) [63,64].

The ctl-1 gene encodes the enzyme catalase, which catalyzes the conversion of H_2_O_2_ to H_2_O and oxygen [65]. Greater expression of ctl-1 could be expected in worms treated with CA or DHCA, where daf-16 was overexpressed taking into account that several genes encoding proteins responsible for antioxidant defenses are included among the target genes regulated by DAF-16, such as the mitochondrial superoxide dismutase SOD-3, the metallothionein homolog MTL-1, and the catalases CTL-1 and CTL-2 [66]. However, it has also been described that the expression of ctl-1 can be regulated independently of daf-16 [67], which might explain the results obtained in the present study.

## 3. Materials and Methods

### 3.1. Standards and Reagents

Caffeic acid (CA) was purchased from Acros Organics (Fisher Scientific, Madrid, Spain), dihydrocaffeic acid (DHCA) was purchased from Alfa Aesar (Karlsruhe, Germany), ampicillin sodium salt, nystatin, agar, yeast extract, fluorodeoxyuridine (FUdR), 2′-7′dichlorofluorescein diacetate (DCFH-DA), phosphate-buffered saline (PBS), cholesterol, 2,4-dinitrophenylhydrazine (DNPH) and 2-mercaptoethanol were purchased from Sigma-Aldrich (Madrid, Spain). Dimethyl sulfoxide (DMSO) was obtained from Panreac (Barcelona, Spain). SYBR^®^ SelectMaster Mix and high-capacity cDNA reverse transcription Kit were from Applied Biosystems (Fisher Scientific, Madrid, Spain) and Illustra™ RNAspin mini isolation Kit from GE Healthcare (Amersham, UK).

### 3.2. Strains and Maintenance Conditions

*Caenorhabditis elegans* wild type strain N2 and *E. coli* OP50 bacterial strain were obtained from the Caenorhabditis Genetics Center at the University of Minnesota (Minneapolis, MN, USA). Worms were routinely propagated at 20 °C on nematode growth medium (NGM) plates with *E. coli* OP50 as a food source.

Synchronization of worm cultures was achieved by treating gravid hermaphrodites with bleach:5N NaOH (2:1). Eggs are resistant, whereas worms are dissolved in the bleach solution. The suspension was shaken with a vortex mixer during one min and kept a further minute on rest; this process was repeated five times. The suspension was centrifuged (2 min, 9500 g). The pellet containing the eggs was washed six times with an equal volume of buffer M9 (3 g KH_2_PO_4_, 6 g Na_2_HPO_4_, 5 g NaCl, 1 mL 1M MgSO_4_, H_2_O to 1 L) and after removed the supernatant the eggs were resuspended and kept in a small volume of M9.

Caffeic acid and dihydrocaffeic acid solutions were prepared in DMSO at two concentrations (200 mM and 300 mM) and added to the nematode growth medium during its preparation to get a final concentration on the plates of 200 μM or 300 μM. Control plates were also prepared without the phenolic acid but containing the same volume of DMSO (0.1% DMSO, *v*/*v*). Around 100 to 300 μL of the M9 with eggs (depending on eggs concentration) were transferred and incubated on NGM agar plates without (control plates) or with the compound assayed (treatment plates). When the worms reached the L4 stage, they were transferred to new plates with or without the compounds but also containing FUdR at a concentration of 150 μM to prevent reproduction and progeny overgrowth. The worms were transferred every 2 days to fresh plates with FUdR for the different treatments (with or without CA or DHCA) until they reached the day of the assay.

### 3.3. Stress Assays

Oxidative stress in worms was induced by subjecting the animals to 35 °C heat-shock treatment. The nematodes were cultivated in NGM-*E. coli* OP50 plates in the absence and presence of CA or DHCA (200 μM and 300 μM) from the larval stage L1 until the moment they were submitted to thermal stress, for which they were transferred with a platinum wire to agar plates (Ø 35 mm, 20 worms per plate) and switched to 35 °C for 8 h. Subsequently dead and alive nematodes were counted, and the survival rate compared with that in the control assay (nematodes subjected to the same stress conditions but cultivated in the absence of the compounds). The application of the stress was performed in two states of the nematode development: second and ninth day of adulthood. All assays were performed with approximately 300 nematodes per treatment.

### 3.4. Reactive Oxygen Species (ROS) Levels

The accumulation of ROS was evaluated at days 2 and 9 of adulthood in worms cultivated in presence and absence of CA and DHCA. The cellular ROS were quantified by the dichlorofluorescein assay [68]. Briefly, the worms were individually transferred to the well of a 96-well plate containing 75 μL of PBS and then exposed or not to thermal stress (2 h at 35 °C), after which 13 μL of DFCH-DA 150 µM solution in ethanol was added to each well. The acetate groups of DFCH-DA were removed in worm cells, and the released DFCH is oxidized by intracellular ROS to yield the fluorescent dye DCF. The fluorescence from each well was measured immediately after incorporation of the reagent and every 10 min for 30 min, using 485 and 535 nm as excitation and emission wavelengths, respectively. Recording of the DCF fluorescence intensity with time in single worms was used as an index of the individual intracellular levels of ROS. Three independent experiments were performed per treatment, and, for each experiment, ROS measurements were made in at least 24 individual worms. The measurements were performed in a microplate reader—FLUOstar Omega, BMG Labtech (Offenburg, Germany)

### 3.5. Lifespan Assay

Age synchronized young larvae (L1) were transferred to fresh NGM agar plates (96 mm) containing the assayed compound CA (200 µM) or DHCA (300 µM) or without compound (DMSO 0.1%) and grown at 20 °C. From L4 stage (2.5 to 3 days later) and every two days, twenty animals were transferred with a platinum wire to small fresh NGM agar plates (35 mm), also with or without the compound assayed and FUdR to prevent reproduction and to avoid overlapping generations. The survival of the worms was counted over the entire lifespan. Worms were scored as dead if they did not respond to touch stimulus with the platinum wire. Three independent assays were carried out for each compound and at least 100 nematodes were used per assay.

### 3.6. RT-qPCR Analyses

Adult worms of the N2 *C. elegans* strain were treated with or without 200 μM of CA or 300 μM of DHCA for 5 days. The worms were periodically transferred to new plates with the phenolic compound or DMSO and *E. coli* as food. These assays are carried out under normal growing conditions (20 °C). The worms were collected with M9 buffer, centrifuged at 10,000 g 1 min, the pellet was resuspended in 300 µL of M9, and 3.5 µL of 2-mercaptoethanol was added. Total RNA was extracted using RNAspin Mini RNA Isolation Kit (GE Healthcare). In order to maximize cell breakage, in the first stage of the extraction, 10 stainless steel beads (2 mm) were added. The mixture was vortex shaken vigorously and further homogenized in a Thermo Savant FastPrep 120 Cell Disrupter System (Waltham, MA, USA) with a speed of 5.5 m/s and run time duration of 10 s five times. A Nanodrop spectrophotometer was used to quantify the amount of RNA.

cDNA was produced with High Capacity cDNA Reverse Transcription Kits (Applied Biosystems) using a 2 µg of total RNA per reaction. The expression of mRNA was assessed by quantitative real-time PCR, using SYBR green as the detection method. Gene expression data were analyzed using the comparative 2-ΔΔCT method with *act-1* as the normalizer. Nine independent experiments were performed; the dissociation curve was determined to confirm a single amplification. The following gene-specific primers were used: *act-1*: CCAGGAATTGCTGATCGTATG (F) and GGAGAGGGAAGCGAGGATAG (R) [15]; *daf-16*: CCAGACGGAAGGCTTAAACT (F) and ATTCGCATGAAACGAGAATG (R) [69]; *sir-2.1*: GACAAAGAACAGAAAGTACAACCAG (F) and GGAGTGGCACCATCAT-CAAG (R) [70]; *skn-1*: AGTGTCGGCGTTCCAGATTTC (F) and GTCGACGAATCTTGCGAATCA (R) [71]; *sod-3*: CGAGCTCGAACCTGTAATCAGCCATG (F) and GGGGTACCGCTGATATTCTTCCACTTG (R) [72]; *daf-18*: TACGGAACAAGCAATGG (F) and AGTCATCCTTGACGATACCTTT (R) [73]; *hsp-16.2*: CTGCAGAATCTCTCCATCTGAGTC (F) and AGATTCGAAGCAACTG-CACC (R) [69]; *hsf-1*: GAAATGTTTTGCCGCATTTT (F) and CCTTGGGACAGTGGAGTCAT (R) [74]; *ctl-1*: AATGGATACGGAGCGCATAC (F) and TCCTGTTCAGCACCATCTTG (R) [75]. Quantitative PCR (RT-qPCR) was performed at the DNA Sequencing Service of the Nucleus platform of the University of Salamanca, with the BioMark system™ HD (San Francisco, CA, USA).

### 3.7. Statistical Analysis

The statistical analyses were performed using the PC software package SPSS (version 23.0; SPSS Inc., Chicago, IL, USA). ANOVA was applied for multiple comparisons of values to determine possible significant differences between treated and control groups. To analyze survival against thermal stress, contingency tables were prepared, and statistical significance was calculated using the Chi Square Test. In every analysis, significant differences were statistically considered at the level of *p* < 0.05.

## 4. Conclusions

Caffeic acid (CA, 200 μM) and dihydrocaffeic acid (DHCA, 300 μM) induced an increase in the survival rate of *C. elegans* under thermal stress, although, in worms treated with CA, the effect was only significant in older worms (day 9 of adulthood), while, in worms treated with DHCA, this increase was observed both in young (day 2) and old adults. These observations seem to suggest that the ability to protect against stress improves by prolonged exposure to these phenolic acids and/or older worms are more susceptible to their effects. Both acids also showed the ability to prolong mean and maximum lifespan in *C. elegans*, compared to untreated worms. In general, treatments with these acids led to a reduction in ROS concentrations, although not always significant, raising the question of whether variations in the level of reactive species, within the observed small oscillations, are actually enough to explain the improvement in stress resistance produced by these compounds, and also suggesting that other mechanisms could be involved in the biological effects. Gene expression studies conducted by RT-qPCR showed that the favorable effects of CA and DHCA on oxidative stress and longevity could involve the activation of several genes related to insulin/IGF-1 pathway, such as *daf-16*, *daf-18, hsf-1,* and *sod-3,* as well as a sirtuin gene (*sir-2.1*). Especially outstanding was the increase in the expression of the *sod-3* gene, which encodes in *C. elegans* an enzyme ortholog SOD2 in humans with superoxide dismutase activity, which could be expected to facilitate the elimination of radical superoxide.

## Figures and Tables

**Table 1:** Influence of CA (200 µM) and DHCA (300 µM) on mean and maximum lifespan of *C. elegans* under normal growing conditions at 20 °C. The results represent the mean ± SEM (*n* = 3).

Treatments	Mean Lifespan (days)	*p* vs. Control (Log-Rank)	Maximum Lifespan* (days)	*p* vs. Control (ANOVA)
Control	16.4 ± 0.39		26.9 ± 0.63	
CA (200 µM)	19.0 ± 0.47	0.001	30.0 ± 0.72	0.009
DHCA (300 µM)	18.7 ± 0.46	0.001	28.9 ± 0.45	0.014

* Average maximum lifespan of 10% of the longest-lived population.

## Sample Availability

Samples of caffeic and dihydrocaffeic acids are available from the authors.
